# Specific skill training for low-resource countries: A perspective from a broad workforce development project in Afghanistan

**DOI:** 10.7189/jogh.10.020398

**Published:** 2020-10-26

**Authors:** Julia L Weinkauf, Alberto E Ardon, Solen Feyissa, Mohammad Sharif Oria, Carolyn Marie Porta

**Affiliations:** 1 Department of Anesthesiology, University of Minnesota, Minneapolis, Minnesota, USA; 2 Department of Anesthesiology and Perioperative Medicine, Mayo Clinic, Jacksonville, Florida, USA; 3 Office of E-Learning Services, School of Public Health, University of Minnesota, Minneapolis, Minnesota, USA; 4 Department of Anesthesiology, Kabul University of Medical Sciences, Kabul, Afghanistan; 5 School of Nursing, University of Minnesota, Minneapolis, Minnesota, USA

Even with obvious training needs, high quality and effective content can be challenging to develop for educational programs targeting resource-poor environments. Entire academic fields and bodies of literature exist for various approaches to education in high-resource settings, but such literature is nascent for low-resource environments, including our field of Anesthesiology [1]^1^. In this article, we discuss some of the challenges and our approach to developing content and a curriculum for teaching highly technical skills as part of an anesthesiology higher education project in Afghanistan. Our strategy involved using Instructional Design principles and accepted strategies of workshop development but modifying them from the beginning in collaboration with the local providers with the hopes of maximizing local relevance.

Approximately US$132 billion has been spent by the United States on reconstruction (non-warfighting) activities in Afghanistan since 2002 [2]. The University Support and Workforce Development Program (USWDP), funded by the US Agency for International Development (USAID), was a 5-year, 93 million-dollar aid package implemented from 2013 to 2019. Its focus was on improving higher education conditions and workforce capacity in Kabul, Afghanistan. A smaller sub-grant of the project aimed to support health training programs at Kabul University of Medical Sciences (KUMS), the only public health sciences university in Kabul. During the project’s planning phase, KUMS administrators identified anesthesiology as one of the academic departments that would benefit from the support. The University of Minnesota (UMN) was chosen as a sub-award winner to provide this educational support to the Afghan faculty, in collaboration with the Ministry of Higher Education.

The department of anesthesiology at KUMS consists of 7 faculty at 3 hospitals, including Ali Abad, their major teaching hospital. The faculty oversees a four-year undergraduate program to train anesthesia technicians. Upon graduation, these new technicians are then qualified to provide direct clinical care either independently or under supervision of an anesthesiologist in Afghanistan. There is an informal, unfunded anesthesiology training program at KUMS, but Afghanistan does not have a formal public residency program, and the majority of anesthetics are delivered by technicians. Though some of the more senior faculty trained internationally in years past, those opportunities have been unavailable in recent years due to the war; the junior and mid-career faculty were trained in an apprentice-style, informal manner after medical school and a short practice in general medicine. Thus, anesthesiology training overall within the country has suffered from a lack of standardization and formalization. It is natural, then, that during the USWD program, the Afghan faculty had a strong desire to enhance their anesthesiology training and learn highly technical and specific anesthesiology skills during this educational endeavor. The aim of this project, however, was wider in scope. The ultimate mission was to develop the learners’ curriculum, educational skills, and pedagogic capacity in order to improve their undergraduate program. We thus incorporated technical knowledge and skills training into a larger agenda that included curriculum development, teaching and learning techniques, research skills, and instructional design.

The workplan was developed in collaboration with our Afghan anesthesia colleagues by distance communication as well as at an initial in-person planning meeting in Bangalore, India. The goals of this meeting were to get to know each other as much as possible, learn about each other’s clinical and teaching environments, brainstorm together what would be most useful to the Afghan faculty and students and the best ways to accomplish those goals, and, finally, to make hard choices about prioritizing what we had time for. We used instructional design strategies to inform our planning [3], and other evidence-based teaching strategies as possible [4,5].

Our workplan included five in-person large group exchanges over 2 years, in varying locations outside of Afghanistan. We were challenged by the fact that we could not be on-site to learn about their clinical practice or educational environment. Such a visit was prohibited in this specific project due to security reasons. This limitation presented an extreme challenge – one day in the hospital would have made up for hundreds of emails and Skype conversations that were inefficient and sometimes ultimately ineffective.

Another challenge, not unique to our project, was balancing the wishes and perceptions of the Afghan faculty’s needs with our perceptions of what would be useful for them. For example, they requested training and equipment in order to start using trans-esophageal echocardiography in their practice, an advanced clinical imaging technique which we felt was not likely to be suitable to their setting. Another subject in which they requested training was regional anesthesia, an area not currently in their practice, but one which they are required to teach in their training program. Regional anesthesia requires extensive training in specific knowledge and procedural skills before it can be safely implemented into clinical practice, and carries the potential for serious complications to patients.

**Figure:**
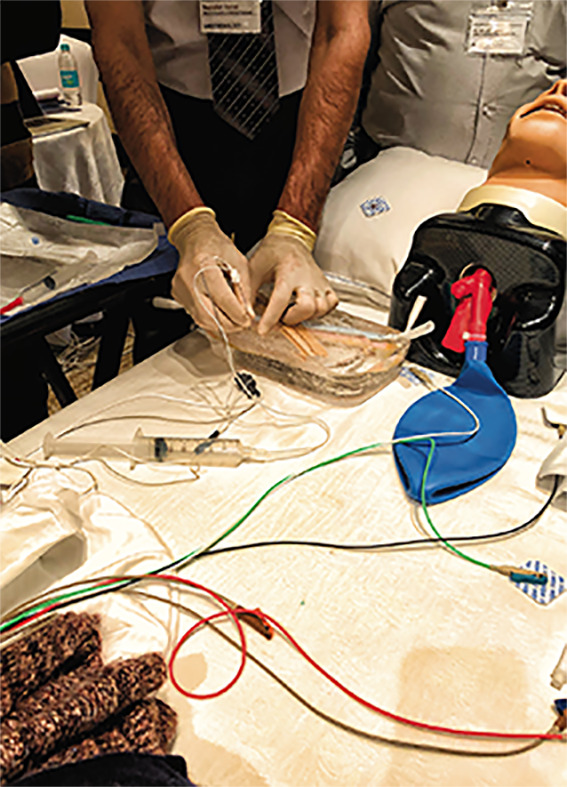
Photo: From Julia Weinkauf's own collection, used with permission.

We had serious hesitations about teaching a topic to them that they could not safely implement clinically back at home. First, it seemed like a potential waste of time – why teach something they couldn’t use, especially when there were so many other clinical areas they did need help with? Furthermore, the lack of emergency equipment specific to complications of regional anesthesia, no ability to do clinical teaching of the procedures on patients, or to gain experience treating the complications, all conspired to make this proposal seem unsupportable. In line with our efforts to develop our workplan collaboratively, however, the Afghan faculty insisted that this was a subject that was part of their curriculum and that they were expected to teach to their students before going on to their respective jobs after graduation. Theirs is the only 4-year undergraduate program in the city, and they felt compelled to offer a level of educational quality that would compete with private programs and maximize skill sets for the job market in Kabul.

This illustrates a larger philosophical question about how to choose material for educational work in limited-resource settings. For example, should health science students be limited in exposure only to the subjects they will be able to practice clinically within their own context? We think not. Though certainly an emphasis should be placed on education that will be practically useful for them to treat patients safely, students in all settings deserve exposure to modern medical knowledge. Indeed, it can be argued that progress is only possible if students have been exposed to what is available and standard in other areas of practice, in order for them to know about and strive for progress.

In this light, we elected to support their request for teaching on this topic and made regional anesthesia the theme of one of our five in-person 1-week exchanges. We taught general concepts on nerve stimulator use and secured two for them to use in their simulation center, which had been established as part of this project. We did the practical teaching on ballistic gel models that could be melted and re-formed for indefinite use. We had a live model patient for landmark identification and positioning. Incorporating pain management into the subject material increased the practical utility of the program as well. The workshop and outcomes are described in detail in the companion article [6].

As with all the exchanges, teaching and learning techniques were woven into the curriculum. During this exchange, the subject was instructional design − a fitting subject, as it had so deeply informed our own approach to the project as a whole. The Afghan faculty learned how to create higher-level (curricula) and lower-lever (individual lesson) teaching materials that were oriented around learning goals, and to create evaluation materials that were mapped to specific learning objectives on both a small and large scale.

By the end of the session, the faculty reported being much better equipped to teach regional anesthesia in a knowledge-based way and did have introduction to the technical skills required. They reported increased confidence and reputation, having been exposed to modern knowledge and technology. Experience in other settings reflects the need for multiple exposures and introductions before highly technical practices are fully established [7]. This session provided one foundational layer that we hope one day might similarly develop into a regional anesthesia practice. More importantly, however, the Afghan faculty left the program with a more robust knowledge base of effective teaching techniques and an improved educational planning skillset, which we believe will ultimately prove to be far more useful in increasing workforce capacity than any one particular clinical skill.
